# Nontuberculous Mycobacteria Immune Reconstitution Syndrome

**DOI:** 10.1155/2014/964612

**Published:** 2014-11-11

**Authors:** J. C. Mogambery, A. Motala, K. Padayachee, C. Jozi, H. Dawood

**Affiliations:** ^1^Infectious Diseases Unit, Department of Internal Medicine, Greys Hospital and University of KwaZulu Natal, Pietermaritzburg, KwaZulu-Natal 3201, South Africa; ^2^Midlands Business Unit, Department of Pathology, National Health Laboratory Service, Pietermaritzburg 3201, South Africa; ^3^Gastroenterology Unit, Department of Internal Medicine, Greys Hospital and University of KwaZulu Natal, Pietermaritzburg, KwaZulu-Natal 3201, South Africa

## Abstract

The prevalence of nontuberculous mycobacteria infection (NTM) in Sub-Saharan Africa is estimated to be less than 1%. NTM is often underdiagnosed or misdiagnosed as tuberculosis in patients who present with immune reconstitution syndrome (IRS) following initiation of antiretroviral treatment (ART). Immune reconstitution syndrome is common in patients who start ART with low CD4 counts and high HIV viral load. Furthermore,* Mycobacterium avium complex* (MAC) commonly infects those with CD4 counts less than 50 cells/mm^3^. Three patients, with low baseline CD4 counts, presenting with NTM following the initiation of antiretroviral treatment are described in this case series. The first patient presented with disseminated NTM two weeks after commencing antiretroviral treatment. Acid fast bacilli were found in the liver, duodenum, and bone marrow and were suggestive of MAC microscopically. The second developed cervical lymphadenitis following the initiation of ART. Lymph node aspirate culture grew NTM. The last patient developed pancytopenia after 3 months of ART. AFB was seen on bone marrow biopsy. Culture of the bone marrow aspirate was suggestive of NTM. All three patients improved on ethambutol, clarithromycin, and rifampicin. NTM may be underdiagnosed in areas with a high TB prevalence and should be actively excluded by culture.

## 1. Introduction

Nontuberculous mycobacterium (NTM) is a ubiquitous environmental organism and the majority of organisms within this family are not pathological in humans [1, 2]. The route of entry in human hosts is by inhalation or ingestion, depending on the species [3]. The most frequently isolated NTM pathogen in Southern Africa is* Mycobacterium avium complex (MAC)* [2]. This organism commonly causes disease in HIV infected patients with CD4 counts less than 50 cells/mm^3^. While lung disease is common in the immunocompetent, MAC often presents with disseminated disease in the HIV infected individual [2].

South Africa has a high prevalence of HIV infected patients, many of whom have very low CD4 counts. A South African study by Pettipher et al. showed a prevalence of* Mycobacterium avium* complex of 10% in 100 HIV infected patients with symptoms suggestive of tuberculosis [4]. Other studies in Africa have not correlated well with this data with prevalence rates of between 0% and 6% in countries like Kenya, Uganda, Malawi, and Zambia [5–9]. As our investigative armamentarium for TB and NTM improves we may find that prevalence estimates increase significantly.

*Mycobacterium avium* complex is the commonest cause of IRS in North America and Europe but there is little evidence to suggest that this disease entity is a problem in Africa [3]. Three cases of nontuberculous mycobacteria immune reconstitution syndrome seen in a South African tertiary hospital are presented.

## 2. Case 1

A 20-year-old HIV infected male with a baseline CD4 count of 17 cells/mm^3^ was referred with cholestatic jaundice. He had recently commenced a fixed dose combination of emtricitabine, tenofovir DF, and efavirenz and was well prior to initiating ART. He developed jaundice two weeks after initiating these drugs. The ART drugs were discontinued by the referring physician as drug induced liver injury was suspected. He was asymptomatic prior to initiating ART. On examination he was emaciated, had yellow sclera and pale conjunctiva, and had a liver span of 18 cm and splenomegaly measuring 5 cm below the costal margin.

His baseline and follow-up blood investigations are shown in Table 1. Hepatitis A, B, and C serologies were negative. The chest radiograph was normal. Ultrasound of the abdomen showed an enlarged “fatty” liver and splenic hypodensities suggestive of granulomas.

The liver ultrasound failed to detect biliary obstruction and a liver biopsy was undertaken to rule out an infiltrative cause of the cholestatic jaundice such as mycobacterial, fungal, or nonbenign infiltration. A gastroscope was performed to investigate the normocytic, normochromic anaemia, and the suspicious lesions noted at gastroscopy were biopsied. In view of the bicytopenia and the absence of a plausible cause, a bone marrow aspiration and biopsy were also performed. The liver biopsy showed multiple acid fast bacilli; however the SD Bioline MPT64 TB Antigen Rapid was negative.

**Figure 1:**
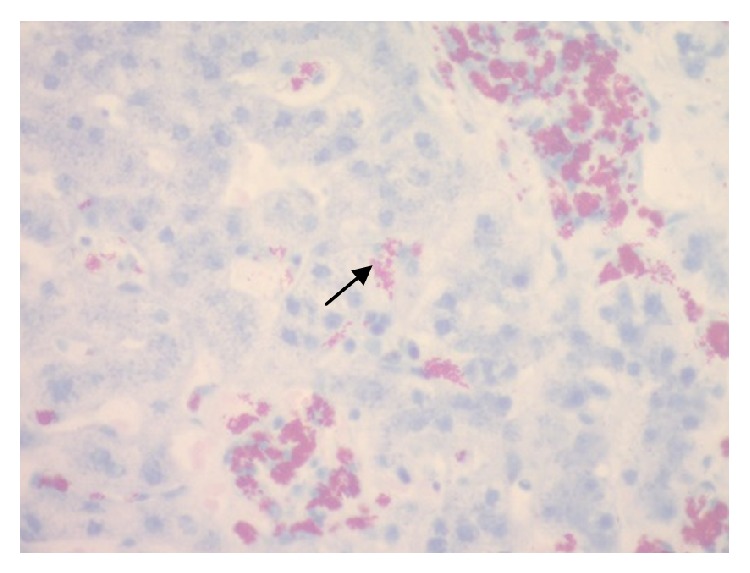
Ziehl-Neelsen staining of hepatic tissue showing numerous and tightly packed AFBs.

**Figure 2:**
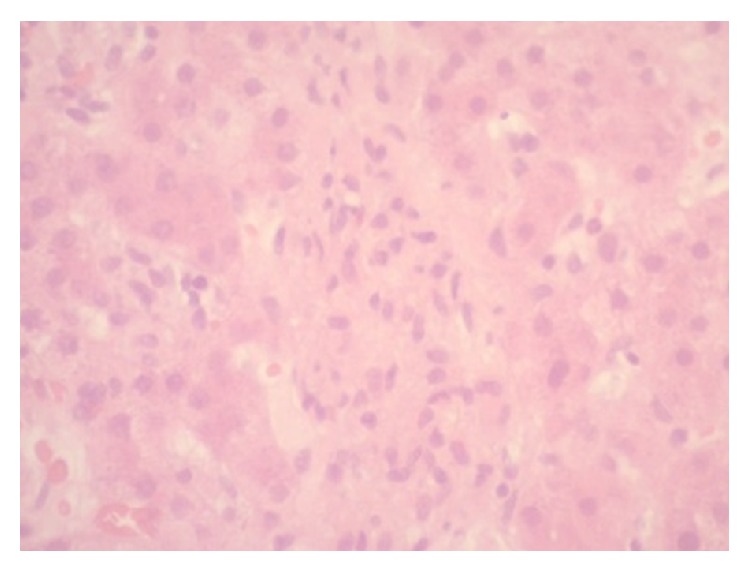
A poorly formed granuloma with surrounding hepatic tissue stained with H&E (Haematoxylin and eosin).

The duodenal biopsy and bone marrow trephine biopsy also showed AFBs. The TB culture was negative for mycobacteria tuberculosis, and microscopy was suggestive of* Mycobacteria avium complex*; however polymerase chain reaction was unable to identify the species.  Figure 1 shows clumping of acid fast bacilli seen in the liver biopsy sample.  Figure 2 shows a poorly formed granuloma commonly seen in nontuberculous mycobacteria infection [3, 10].

He was commenced on rifampicin, isoniazid, ethambutol, pyrazinamide, and clarithromycin because a definitive microbiological diagnosis of TB or NTM was difficult to attain. After a two-month period of treatment, the patient showed significant clinical improvement.

The baseline HIV viral load was not done; however his viral load at 2 weeks of ART and following discontinuation of the drug for suspected drug induced hepatitis was 227 226 copies/mL. A HIV viral load, after 5 months of antiretrovirals, in June 2014 was 756 copies/mL.

## 3. Case 2

A 26-year-old female commenced combination ART in February 2013 with a baseline CD4 count of 63 cells/mm^3^. She had a productive cough and constitutional symptoms, in February 2013, and Ziehl Neelsen stain showed AFB on a sputum sample. Sensitive mycobacteria tuberculosis was detected on GeneXpert MTB/Rif of expectorated sputum. She was commenced on TB treatment and completed a six-month regimen of rifampicin, isoniazid, ethambutol, and pyrazinamide. After completing TB treatment she developed right sided anterior cervical lymphadenopathy, right upper quadrant abdominal pain, vomiting, and nausea. Fine needle aspiration of the lymph node revealed pus that contained AFBs. Drug resistant TB was the initial consideration and the pus was sent for TB culture.

TB culture failed to grow* Mycobacteria tuberculosis*; however NTM was detected. The patient was commenced on rifampicin, ethambutol, and clarithromycin, and the lymphadenopathy resolved without further surgical intervention.

She did not have a baseline HIV viral load but she responded very rapidly to ART and had an undetectable viral load within 5 months of treatment. At one-year follow-up her HIV viral load was undetectable, and her CD4 count was 231 cells/mm^3^. The diagnosis of mycobacteria tuberculosis, made after the initiation of antiretroviral treatment, was probably due to nontuberculous mycobacteria immune reconstitution syndrome or TB/NTM coinfection. The NTM infection was not associated with an increase in viral load or a decline in CD4 count; therefore it is likely that this patient had dual TB/NTM associated IRS, rather than a new opportunistic infection. Serial ART monitoring blood results and TB investigations are presented in Table 2.

## 4. Case 3

A 31-year-old male was admitted with biopsy confirmed cutaneous histoplasmosis. He had developed plaques a few months prior to commencing ART. His baseline CD4 count was 29 cells/mm^3^ in August 2013. A viral load and CD4 count at 3 months of ART were 580 copies/mm^3^ and 278 cells/mm^3^, respectively. He was referred for investigation of pancytopenia.  Table 3 shows his baseline blood results. A bone marrow aspiration as well as biopsy was undertaken to investigate for fungal infiltration of the bone marrow but the biopsy showed AFB. He was commenced on rifampicin, isoniazid, ethambutol, and pyrazinamide. A day later the SD Bioline MPT64 TB Antigen Rapid result was negative. Clarithromycin was added to the TB treatment to effectively treat the disseminated nontuberculous mycobacteria infection. Culture revealed NTM but PCR failed to determine the species.

At his 6-month follow-up visit he had a normal full blood count. Follow-up blood results are captured in Table 2. He continues his TB treatment together with clarithromycin and ART.

## 5. Discussion

Immune reconstitution syndrome (IRS) is an inflammatory phenomenon that occurs following the initiation of ART in patients with very low CD4 counts [2]. Patients present with paradoxical worsening of an existing infection or unmasking of a new infection or inflammatory condition [3]. IRS is characterised by a robust immunological and virological response to the ART [3]. Several infections, autoimmune conditions, inflammatory disorders, and malignancies have been described as causing IRS [2].

Tuberculosis is a common manifestation of IRS in South Africa but NTM, especially* Mycobacteria avium complex*, may be underdiagnosed or misdiagnosed as TB [10]. The symptoms of weight loss, night sweats, and fever are common to both infections [3].

*Common Acid Fast Pathogens Found in HIV Infected Hosts*. Consider the following:

- all mycobacteria including* M. tuberculosis, M. leprae, and M. avium-intracellulare,*
- *Actinomyces nocardia,*
- *Cryptosporidium parvum,*
- *Isospora belli.*

NTM IRIS may manifest as lymphadenitis, pulmonary infiltrates, intra-abdominal lymphadenopathy, or hepatosplenomegaly. Other uncommon foci of infection are bone, skin, and urogenital tract [1, 2]. The route of entry of NTM is often oral; therefore diarrhoea and abdominal pain may be the presenting complaint and differentiating symptoms [3]. Common laboratory findings are normocytic normochromic anaemia and a raised alkaline phosphatase [11].

Treatment includes ethambutol and clarithromycin [11]. Rifabutin is often included to decrease bacterial burden. A three-drug regimen has proven to decrease relapse rates [2]. Rifabutin is not easily available in the public sector in South Africa and is very expensive. For these reasons rifampicin is the preferred alternative. Treatment for nontuberculous mycobacteria is often prolonged, up to 18 months [3]. Use of these agents together with ART is often associated with drug interactions and dose adjustments may be required.

Prophylaxis with rifabutin is indicated in patients with CD4 counts less than 50 cells/mm^3^. This is currently not recommended in South Africa because of the high prevalence of tuberculosis and the increasing incidence of drug resistant TB. Azithromycin has been compared to rifabutin in randomized control trials and has been shown to be more efficacious with a slightly higher rate of adverse events [12].* Mycobacteria avium* complex exposed to azithromycin in prophylactic doses may develop resistance to both azithromycin and clarithromycin and should be therefore used with caution [12].

**Figure 3:**
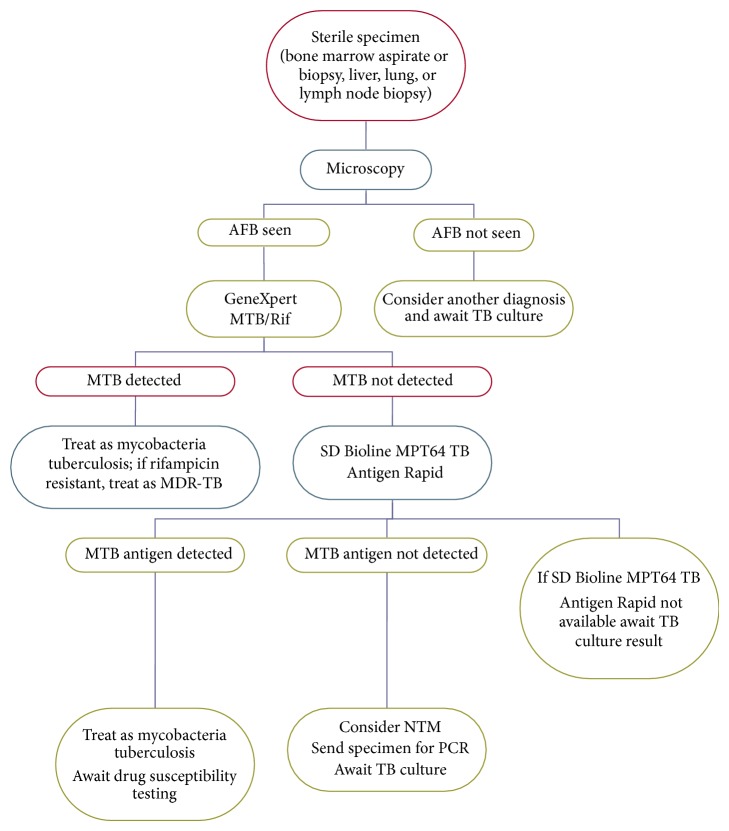
Proposed diagnostic algorithm for NTM.

Due to the recent implementation of GeneXpert MTB/Rif for the diagnosis of TB in South Africa the prevalence of NTM may increase. Prevalence studies are needed as GeneXpert MTB/Rif and SD Bioline MPT64 TB Antigen Rapid have shown that all acid fast bacilli need not be identified as mycobacteria tuberculosis [2]. Culture results should be sought in TB suspects with GeneXpert negative, AFB positive results and patients who fail to respond to a first line tuberculosis regimen or remain AFB positive despite completing TB treatment. We propose the following diagnostic algorithm in Figure 3 as a stepwise approach in the diagnosis of extrapulmonary NTM.

Speciation is often unavailable in resource limited countries. We recommend that treatment for* Mycobacterium avium* complex be commenced without delay in HIV infected patients with CD4 counts less than 100 cells/mm^3^, where AFB is seen on microscopy of a sterile sample and MTB is not detected on GeneXpert.

## 6. Points to Remember

- NTM is an increasingly common opportunistic infection affecting HIV infected patients in Southern Africa.
- It should be considered in patients who present with a paradoxical worsening or new onset of symptoms after commencing ART, despite being on TB treatment and a low baseline CD4 count.
- Diagnosis is often difficult as the presentation is similar to tuberculosis and can only be made by TB culture.
- SD Bioline MPT64 TB Antigen Rapid is a useful diagnostic tool and helps to differentiate between MTB and NTM.

## Figures and Tables

**Table 1:** Showing baseline and follow-up blood results.

	Baseline blood results	Follow-up blood results	
Date	01/14	03/14	06/14
Haemoglobin (g/dL)	7.2	6.4	8.4
Mean cell volume (fL)	91	84	85
Leucocytes (cells/L)	5.7 × 10^9^	6.4 × 10^9^	8.9 × 10^9^
Erythrocytes (cells/L)	1.4 × 10^12^	2.42 × 10^12^	3.5 × 10^12^
Platelets (cells/L)	103 × 10^9^	246 × 10^9^	585 × 10^9^
Bilirubin (umol/L)	196	140	66
ALT (U/L)	58	242	46
AST (U/L)	153	140	86
GGT (U/L)	1046	2552	835
ALP (U/L)	2074	2206	1859
CD4 count (cells/mm^3^)	17		79
HIV viral load (copies/mL)	227 226		756

**Table 2:** Showing ART monitoring blood results and TB microbiology results.

	At initiation of ART	Follow-up blood results		
Date	02/2013	08/2013	09/2013	03/2014
Sputum microscopy	Not done	AFB seen^+++^	Scanty AFB	No AFB seen
Sputum GeneXpert	Not done	MTB detected, sensitive to rifampicin	Not used for follow-up	Not used for follow-up
Lymph node aspirate				
Microscopy				AFB seen
TB PCR				MTB not detected
Culture				NTM isolated, unable to speciate
CD4 count (cells/mm^3^)	63	172	201	231
HIV viral load (copies/mL)		124	Not detected	Not detected

**Table 3:** Showing baseline and follow-up blood results.

	One month after commencement of ART	Admission blood results	Follow-up blood results	
Date	09/2013	11/2013	03/2014	9/2014
Haemoglobin (g/dL)	9.2	6.4	9.4	13.4
Leucocytes (cells/L)	4.2 × 10^9^	1.6 × 10^9^	4.3 × 10^9^	5.2 × 10^9^
Erythrocytes (cells/L)	3.3 × 10^12^	1.98 × 10^12^	2.58 × 10^12^	3.9 × 10^12^
Platelets (cells/L)	199 × 10^9^	48 × 10^9^	311 × 10^9^	472 × 10^9^
Bilirubin (umol/L)		9	5	6
ALT (U/L)		50	17	21
AST (U/L)		55	28	25
GGT (U/L)		217	188	118
ALP (U/L)		304	165	90
CD4 count (cells/mm^3^)	29	278		409
HIV viral load (copies/mL)	13617	112		Not detected
